# Case of Paraplegia

**Published:** 1828-01

**Authors:** Charles Thomas

**Affiliations:** Devonport.


### PARAPLEGIA.

*Case of Paraplegia.* By Charles Thomas, M.D. Devonport.

John Shapcot, at the time of his death forty-three years of age, (twenty-two of which had been spent in the navy,) tall, robust, and well proportioned; till his last illness recollected no interruption to the full enjoyment of health, except an attack of fever in India fifteen years since, and of rheumatism, which, in the winter of 1824-5, confined him to the house four months.

The circumstances of the case, as they occurred before I visited him, I have obtained from the patient himself, his wife, and a respectable surgeon, under whose care he was placed.

Towards the end of last March, after sleeping several nights in a very damp room, the first symptom which attracted his attention was a pain of the left side, sometimes extending across the lower part of the back. In a day or two, increasing weakness of the legs was felt, and some degree of numbness. The bowels became constipated, and a difficulty in voiding his urine was perceived. This last symptom soon amounted to complete retention; and not till it had existed for four days was any professional assistance sought. There had been no sort of complaint of the head.

The treatment during the month of April consisted chiefly in regularly relieving the bladder by the catheter, and the constipation by frequent enemas, and occasional moderately active purgatives. The spine was also frequently rubbed with a powerful stimulating liniment. In the course of three weeks, the numbness and weakness of the legs had nearly left him; the urine could be expelled so well by a voluntary effort that the use of the catheter seemed no longer necessary; and a regular state of the bowels was in a great degree restored. But these promising appearances were of short duration. By the beginning of May the symptoms returned more intensely than ever; and on the 5th of that month, when he became a patient of the Devonport Dispensary, he was completely paraplegic. From the lower lumbar vertebræ to the toes, there was an entire abolition of sensation and motion. Severe pricking and pinching of the skin made no impression whatever. The vesical and intestinal excretions were wholly suspended. There was no complaint of the head; nor, indeed, pain any where, but a state of general uneasiness, and almost total deprivation of sleep. Appetite continued good, as it had been from the commencement of the attack. For upwards of a week the treatment was similar to that before described, with the addition of repeated and extensive blistering along the spine, and the administration of an opiate every second or third night. From the latter, through all the subsequent stages of his illness, he never failed to derive great refreshment.

On the 14th of May, extensive redness began to show itself on the sacrum. By the 20th, the integument sloughed, leaving a circular ulcerated surface, five inches in diameter. He now felt weaker, with some failure of appetite. Sulphate of Quina was prescribed, to which, in a few days, Carbonate of Ammonia was added, at the recommendation of my colleague, Dr. Magrath.

June 1st.—The ulcer had never spread beyond its original dimensions, or assumed any other than a favorable aspect. Indeed, it now began to cicatrise about the edges. There was also an evident amendment in the disease. He could raise his legs a little in the bed, and perceive some feeling in them.

10th.—This encouraging change had gone on daily improving. Motion and sensation were so far restored that, with slight assistance, he could bear his legs on the floor. The catheter was still used, but he could make a small quantity of urine several times in the day without it. For some time a great deal of mucus had been discharged with the urine, and now a considerable quantity of pus was discovered. But there had been no pain in the bladder since the period of first employing the catheter.

17th.—This morning the poor patient told me, as soon as I entered the room, that the urine had suddenly come away during the night by the rectum. This occurrence, though not altogether unexpected, created in my mind no little regret; for the paraplegia might then be said to be entirely gone.

Under this condition of diseased bladder he continued to linger till the time of his death, which happened on the 21st of July.

It was a source of great disappointment to me, that a case of so much interest could not be further elucidated by an examination after death. But his widow was inflexible in her opposition to it. Still, without this evidence, it affords us enough of a different kind, from which several important inferences may be deduced. It goes far, though the evidence upon which it rests is but negative, to confirm the admirable observations of Dr. BURDER, in the Journal for May, where a pathology of paraplegia, though supported by the venerable name of BAILLIE, and adopted by a large portion of the profession, is called in question with so much research, candour, and sound reasoning, as to reflect a high degree of credit on the author.

In the present instance, through the whole course of the disease, *not a single uneasy sensation was felt in the head,* as the patient unequivocally declared, when repeatedly interrogated on that point, while he distinctly stated that pain of the *back* was frequent, and sometimes severe.

Another and a more remarkable incident, is the coincidence between the existence of an extensive ulceration on the sacrum and lower lumbar vertebræ, and the gradual disappearance of all the paraplegic symptoms. Have we not here a strong presumption, not only of the spinal marrow being the seat of the disease, but also the important practical fact that we are often satisfied with issues occupying a space too limited to give them a due degree of efficiency ?

The diseased condition of the bladder, and the causes of it, in this and similar cases, form a curious subject of pathological investigation. The extremely distended state of this viscus for four days before any professional relief was sought might have induced an ulcerative process in its coats, which ultimately led to their disruption. Or are we to suppose that, the nervous power of the part being lost, the urine became decomposed, and continued to act as an irritant on the mucous surface to the entire disorganization of the bladder, in accordance with what I believe has been observed in experiments made on animals ?

Dr. Burder, after quoting a passage from a letter he had received last December, makes this remark:—"Thus, the powerful authority of Dr. Abercrombie cannot, with any propriety, be now advanced as confirming the cerebral origin of paraplegia." Nor could it before have been so, if we look to the tenor of his valuable observations on this subject; for whatever importance may have been affixed to the four cases adduced as proof of such an opinion being entertained by that distinguished pathologist, yet he expressly says, "Paraplegia *generally* depends upon disease in the spine. It is sometimes, however, produced by affections of the head, as indurations of the cerebellum, or tumors about the medulla oblongata."*

I cannot conclude this communication without adverting to another case, very analogous to what I have related, and which leads to the same deductions respecting the proximate cause of paraplegia. A gentleman in this county sunk under that disease, after lingering a year. During the course of it there was severe and almost constant pain of the back, but none in the head. After death, the brain exhibited no traces whatever of diseased structure: the spinal cord was not examined. P.65.

*Devonport; Oct.* 15, 1827.

### CRITICAL ANALYSES.

* Edinburgh Medical Journal, January 1819.

